# Multi-Layer Laminate of Fibreglass Thermoplastic Composite Reinforced with Fused Filament Fabrication TPU Layers

**DOI:** 10.3390/polym17192622

**Published:** 2025-09-28

**Authors:** Ana Paula Duarte, Pedro R. da Costa, Manuel Freitas

**Affiliations:** 1 Atlântica—Instituto Universitário, Fábrica da Pólvora de Barcarena, 2730-036 Barcarena, Portugal; apduarte@uatlantica.pt (A.P.D.); pcosta@uatlantica.pt (P.R.d.C.); 2 CERENA—Centro de Recursos Naturais e Ambiente, Instituto Superior Técnico, Av. Rovisco Pais, nº 1, 1049-001 Lisboa, Portugal; 3 C-MAST—Centre for Mechanical and Aerospace Science and Technologies, Universidade da Beira Interior, Convento de Sto. António, 6201-001 Covilhã, Portugal

**Keywords:** thermoplastic composites, thermoplastic polyurethane elastomer, fused filament fabrication, polypropylene, fibreglass, interlayer toughening

## Abstract

Thermoset fibre-reinforced composites are widely used in high-end industries, but a growing demand for more sustainable and recyclable alternatives conveyed the research efforts towards thermoplastics. To expand their usage, new approaches to their manufacture and mechanical performance must be tackled and tailored to each engineering challenge. The present study designed, manufactured and tested advanced multi-layer laminated composites of thermoplastic polypropylene prepreg reinforced with continuous woven fibreglass with interlayer toughening through thermoplastic polyurethane elastomer (TPU) layers manufactured by fused filament fabrication. The manufacturing process was iteratively optimized, resulting in successful adhesion between layers. Three composite configurations were produced: baseline glass fibre polypropylene (GFPP) prepreg and two multi-layer composites, with solid and honeycomb structured TPU layers. Thermal and mechanical analyses were conducted with both the polyurethane elastomer and the manufactured laminates. Tensile testing was conducted on additively manufactured polyurethane elastomer specimens, while laminated composites were tested in three-point bending. The results demonstrated the potential of the developed laminates. TPU multi-layer laminates exhibit higher thermal stability compared to the baseline GFPP prepreg-based composites. The addition of elastomeric layers decreases the flexural modulus but increases the ability to sustain plastic deformation. Multi-layer laminate composites presenting honeycomb TPU layers exhibit improved geometric and mechanical consistency, lower delamination and fibre breakage, and a high elastic recoverability after testing.

## 1. Introduction

Thermoplastic composites have attracted growing interest in their research and applicability due to their promising balance of mechanical performance and processability. They are increasingly used in sectors such as aerospace and automotive, offering advantages such as high specific strength, excellent corrosion resistance, recyclability, low density, good vibration damping, and infinite shelf life [1,2]. There is a vast variety of thermoplastic polymers in use with fibre reinforcement. In aerospace, carbon polyetherketoneketone (PEKK) and polyphenylene-sulphide (PPS) have already been used in structural aviation components [3]. Polypropylene (PP) is extensively used with and without fibre reinforcement in the automotive industry [4]. However, in high load structural components, thermoset composites are still the standard. The expansion of thermoplastic composites into complex, high-end engineering applications calls for new manufacturing and performance strategies to better tailor their properties. To strengthen the competitiveness of thermoplastic composites over traditional non-recyclable thermoset composites, several research directions are being explored. Among these, interlayer toughening has emerged as a promising strategy to enhance interlaminar strength, a known weak point for laminate composites [5].

The interlaminar regions are thin polymer-rich zones with relatively weak mechanical properties. Under load, these regions are subjected to stresses caused by mechanical and thermal anisotropic differences between plies [6]. Their behaviour depends on the mechanical properties of the matrix phase as well as on the interaction of the two subsequent plies affected by the fibre architecture, orientation, and lamination sequence [5,6]. Toughening the interlaminar region is an interesting strategy to bring substantial value to thermoplastic composites by improving damage tolerance, delamination resistance, and strength-to-weight ratio [5,6]. Within interlayer toughening approaches we find several solutions, such as particle/filler-based solutions [7]; film interleaving methods [8]; nanofibrous interlayer toughening [9], and interlaminate reinforcement layers [10].

Interlaminate layer reinforcement is a proven solution that enables high tailoring abilities towards complex mechanical properties such as fracture toughness and slow crack propagation. Examples include metal and polymeric interlayers such as aluminium (GLARE) [10] and toughened resin films [8]. Other research approaches have shown that thermoplastic polymers applied to resin-based composites can enhance delamination toughness and impact performance [11,12].

Across all thermoplastic manufacturing methods, additive manufacturing (AM) has emerged in recent years as a transformative technology across multiple industries, enabling new approaches to general design, material design, prototyping, and production [13]. Among the various additive techniques, fused filament fabrication (FFF) has gained prominence due to its accessibility and versatility. FFF is already recognized for its competitive and wide range of equipment, its ability to produce complex 3D components with good mechanical properties, and its broad selection of available materials [14,15]. Since the emergence of FFF, acrylonitrile butadiene styrene (ABS) and polylactic acid (PLA)-based thermoplastics have been the most widely studied [16]. FFF adaptability allows for continuous expansion on material design, from low- to high-grade engineering materials such as polyether ether ether ketone (PEEK) [17], polypropylene with and without short glass fibre reinforcement [18], continuous carbon fibre-reinforced PLA composites [19], and viscoelastic materials, namely thermoplastic elastomers (TPEs).

TPEs are defined as a branch of elastomeric polymers that, unlike vulcanized rubber, can be processed and recycled like thermoplastics. Most TPEs are phase-separated systems with rigid and elastomeric phases. These phases are often chemically linked through block or graft polymerization, although in some cases, a fine dispersion of phases suffices [20]. This results in a unique non-linear viscoelastic stress–strain mechanical response together with a very high deformation degree before failure, ideal for energy-absorbing and damage-mitigating applications [21,22].

Among TPEs, the thermoplastic polyurethanes (TPUs) are known for their high ductility, excellent abrasion resistance, and good biocompatibility. TPUs offer a wide range of mechanical properties by changing the soft to hard segment ratio. This ratio largely determines TPUs’ elastic and low-temperature behaviour; consequently TPUs can exhibit shore hardness from soft 70A to hard 74D, tensile strength from 20 to 50 MPa, and elongation at break between 400 and 700% [20,21,23]. This versatility positions TPUs as a unique bridge between rubber-like and structural polymers. When combined with the design flexibility of FFF, TPUs present new opportunities for creating structural components with tailored mechanical behaviour for various fields such as non-pneumatic tyres [24] and medical devices [25].

This study investigated interlayer toughening of glass fibre-reinforced thermoplastic polypropylene (GFPP) laminates using additively manufactured TPU layers in both bulk and honeycomb configurations. FFF was chosen among the various AM techniques due to its accessibility and versatility in layer design, enabling tailored TPU layers for the intended laminate composite under study. Tensile testing was conducted with bulk and honeycomb TPU specimens under various FFF printing parameters. Followed by TPU mechanical characterisation, three combinations of GFRP composites, two with TPU interlayer reinforcement—bulk and honeycomb shaped—were tested under a three-point bending test. To further understand the toughening mechanisms and interfacial interactions, surface morphology and thermal analyses were also carried out.

## 2. Materials and Testing Procedure

TPU specimens were first manufactured and characterized in tensile testing to assess their suitability as interlayer reinforcements. Subsequently, GFPP laminates were produced, both with and without TPU interlayers, and tested under three-point bending.

### 2.1. Raw Materials and Specimens Manufacture

#### 2.1.1. TPU

TPU specimens and TPU interlayer reinforcing layers were manufactured using Creality Ender 3 S1 (Creality 3D Technology Co., Ltd., Shenzhen, China) equipped with a direct drive extrusion system coupled with a 0.4 mm nozzle. The material used was TPU95A Ultrafuse from BASF (BASF 3D Printing Solutions GmbH, Heidelberg, Germany). Table 1 summarizes the filament’s key mechanical and thermal properties, as specified by the manufacturer.

The FFF printing of elastomeric materials like TPU has proven challenging due to their flexibility and sensitivity to process settings. Extrusion temperature strongly affects print quality and mechanical performance [27]. To characterize its effect, TPU was printed at three temperatures, 215 °C, 225 °C, and 235 °C, covering and slightly exceeding the processing range (Table 1) to observe variations in mechanical and thermal behaviour. PrusaSlicer software version 2.9.0 was used to generate all G-code files for FFF. All samples were manufactured with a 0.2 mm layer height; a printing speed of 10 mm/s; no top or bottom layers; a filament extrusion multiplier of 1.4; and a heat bed temperature of 60 °C.

**Figure 1:**
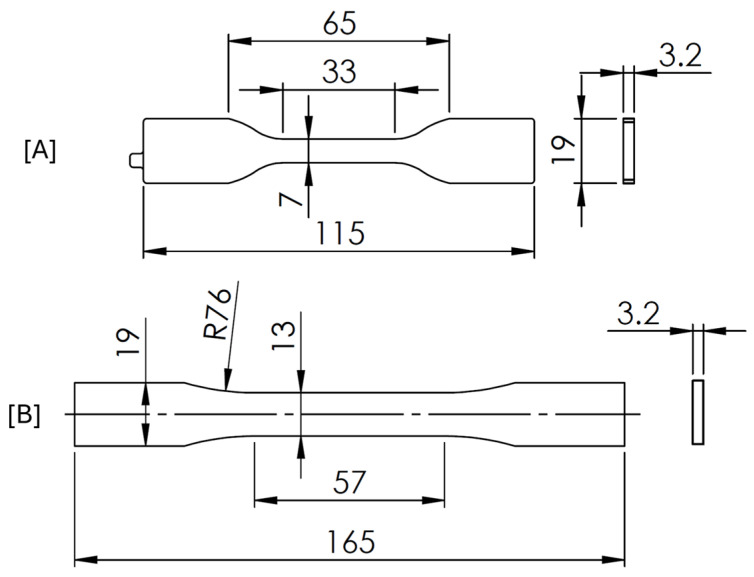
TPU tensile specimens following ASTM D638: (**A**) Type IV for bulk; (**B**) Type I for honeycomb cellular structure.

TPU was tested in bulk and with the cellular honeycomb structure intended for interlayer reinforcement. Specimens were designed according to ASTM D638 ‘Standard Test Method for Tensile properties of Plastics’ [28]. The standard provides multiple specimen geometries: Type IV (Figure 1A) was used for bulk TPU, as it is recommended for elastomeric materials, while Type I (Figure 1B) was chosen for cellular samples. The change in specimen type was made to take advantage of its larger constant cross-sectional area, which allows more cells to be engaged under load. Previous studies have shown that specimens with fewer cells tend to underpredict stiffness and effective modulus compared to larger, more representative samples [29,30]. The change in specimen type will therefore improve the mechanical response, accuracy, and consistency representation of cellular structure under load. A total number of 15 sets of specimens were tested: 9 sets of bulk Type IV (Figure 1A) specimens and 6 sets of honeycomb cellular Type 1 specimens (Figure 1B).

Since FFF manufactures by placing filaments at each layer, the results have proven to be anisotropic on both the rigidity and strength in accordance with the alignment of filament direction to load. Several published studies in tensile and compressive testing have shown the anisotropic behaviour both for thermoplastic and elastomeric FFF printed materials [17,21,31]. The proven anisotropic behaviour needs, therefore, to be characterized. Accordingly, specimens were manufactured with different filament alignment to load. Bulk specimens were manufactured with filaments aligned (0°), transverse (90°), and at a (45°) angle in respect to the load direction. A total of 9 sets of bulk Type IV (Figure 1A) specimens were manufactured, combining all six parameters under study: 3 extrusion temperatures (215 °C, 225 °C, and 235 °C) and 3 load filament alignments (0°, 45°, and 90°).

**Figure 2:**
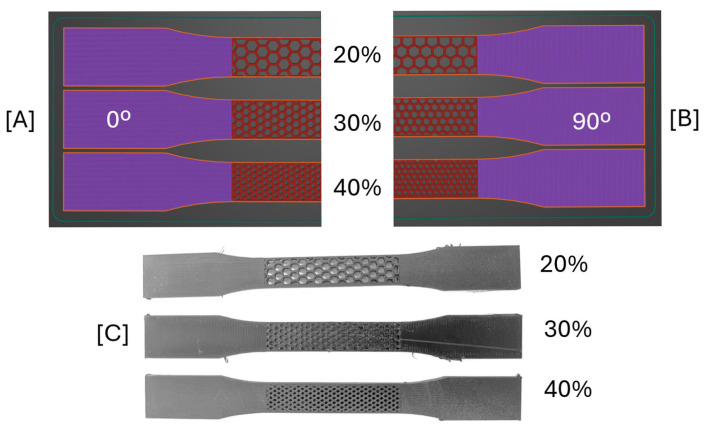
Illustration of the three relative densities tested for (**A**) an aligned load (0°) and (**B**) a load transverse (90°) to the TPU specimens. (**C**) Photo of honeycomb TPU Type I specimens with load aligned with the structure (0°).

The honeycomb structure is established as an in-plane orthotropic cellular structure. To characterize such behaviour, two different directions were tested: cell separation wall aligned with the load (0°) and transverse to the load (90°). Additionally, three different relative densities were tested (20%, 30%, and 40%). A total of 6 sets of honeycomb Type I (Figure 1B) specimens were manufactured, combining 3 relative densities and 2 load direction parameters. The honeycomb structure was applied only in the constant cross-section area region of the specimen with 57 mm in length. Figure 2 illustrates the 0° and 90° difference for the three relative densities tested.

#### 2.1.2. Composite Laminates

Glass fibre-reinforced thermoplastic polypropylene (GFPP) laminates manufactured with and without FFF TPU are now discussed. The employed prepreg was the WG1-PP-700 black weave twill 2/2, by COMFIL (Gjern, Denmark). Base properties are presented in Table 2.

In all manufactured laminates, three twill 2/2 prepreg layers were applied, with TPU interlayer reinforcements of 0.4 mm thickness placed between the prepreg plies.

Three composite configurations were produced: a baseline and two multi-layer composites. Table 3 details the different manufactured laminates, abbreviations, and representative layer sequences. The honeycomb cellular structures were produced with 25% relative density infill, chosen as an intermediate balance between mechanical contrast and manufacturing feasibility: low enough to ensure a measurable difference in flexural response, yet high enough to provide clear structural continuity and observable effects during testing and microscopic analysis.

**Figure 3:**
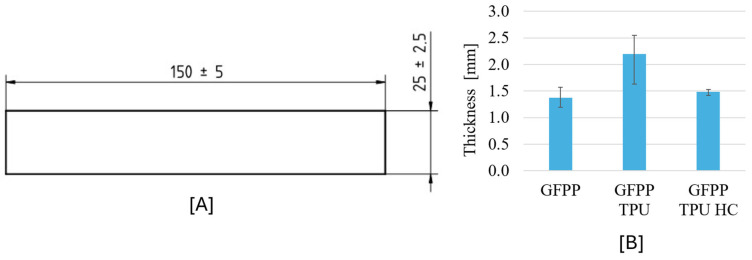
(**A**) Composite flexural specimen geometry (mm) and (**B**) measured average thickness for different specimen configurations.

All GFPP laminates were manufactured in a vacuum bag with a curing temperature of 200 °C. After reaching the curing temperature, they were gradually cooled to room temperature with the oven door open and later machined by CNC followed by edge polishing. From the laminate plates, three-point bending specimens were machined. Figure 3 shows the manufactured geometry according to the ASTM D790-17 standard and the final measured thickness of the three composite configurations.

From laminate thickness measurements (Figure 3B), an interesting conclusion was attained. The composite with TPU honeycomb layers (GFPP-TPU-HC) demonstrated significantly less thickness variability. This might indicate that the honeycomb layer can help to achieve a more uniform composite laminate under a vacuum bag. The mechanical tests reinforce this conclusion, as GFPP-TPU-HC presented an overall equal or even lower variability on all results. This behaviour could be attributed to the high deformation and adaptability of the TPU honeycomb layer, enabling a more uniform pressure distribution to be applied to the laminate across the curing process. The higher contact area of the honeycomb structure could also help guarantee good layer adhesion in manufactured samples, as later perceived in mechanical testing and microscopic analysis.

### 2.2. Mechanical Testing Methodology

TPU FFF specimens were subjected to tensile testing while all manufactured composite laminates were subjected to three-point bending.

#### 2.2.1. Tensile Testing

TPU was tested in bulk and with the cellular honeycomb structure intended for interlayer reinforcement. Tensile testing was conducted on a Universal Testing Machine (Instron 5966, Instron, Norwood, MA, USA) equipped with a 10 kN load cell. All tensile specimens were tested at a speed of 10 mm/min. Given the TPUs’ complex non-linear response to load [20], to mechanically characterize their strength and stiffness in tensile testing, three parameters were computed: the initial elastic modulus (*E_i_*) between 1% and 5% strain, elongation at break (fractured strain, ε_f_), and tensile strength (rupture stress, S_r_). The initial elastic modulus *Ei* was determined as the mean slope of the nominal stress–strain curve between 1% and 5% strain, a method widely adopted in the literature to describe the elastic response of elastomeric materials [21,22,23].

#### 2.2.2. Three-Point Bending Testing

All three-point bending tests were also conducted with a Universal Testing Machine (Instron 5966) equipped with a 10 kN load cell and a 100 mm distance between supports. The standard ASTM D790-17 ‘Standard Test Methods for Flexural Properties of Unreinforced and Reinforced Plastics and Electrical Insulating Materials’ [33] outlines the method for determining the flexural properties in three-point bending of unreinforced and reinforced plastics.

### 2.3. Thermal Characterization Procedure

Thermal analysis was conducted on both TPU and composite laminate specimens.

Differential scanning calorimetry (DSC) was used to characterize the TPU filament (prior to FFF) and after printing at 215 °C, 225 °C, and 235 °C. Post-printed samples were extracted from the tip made in Type IV geometry (visible in Figure 1A). Measurements were carried out using a HITACHI STA 7200 (HITACHI, Tokyo, Japan) in the range of room temperature to 220 °C with a heating rate of 10 °C/min using a nitrogen flow rate of 50 mL/min. Melting temperatures (T_m_) and crystallization temperatures (T_c_) were determined. A second DSC analysis at lower temperatures was conducted from −90 °C to 75 °C to determine the glass transition temperature (T_g_).

The laminate composites were analysed by differential thermal analysis (DTA) and thermogravimetric analysis (TG), carried out again in the HITACHI STA 7200. Samples ranging from 7 to 10 mg were taken from all composite specimens. The trials were carried out from room temperature (25 °C) to 550 °C (heating rate 10 °C/min) using alumina crucibles and a nitrogen atmosphere (200 mL/min) to avoid the occurrence of oxidation reactions. From these analyses, fibre/polymer mass fraction, the degradation temperatures of PP and TPU, and the thermal stability of all three manufactured laminates were determined.

## 3. Results Analysis

### 3.1. Thermal Characterization

#### 3.1.1. TPU Differential Scanning Calorimetry Analysis (DSC)

**Figure 4:**
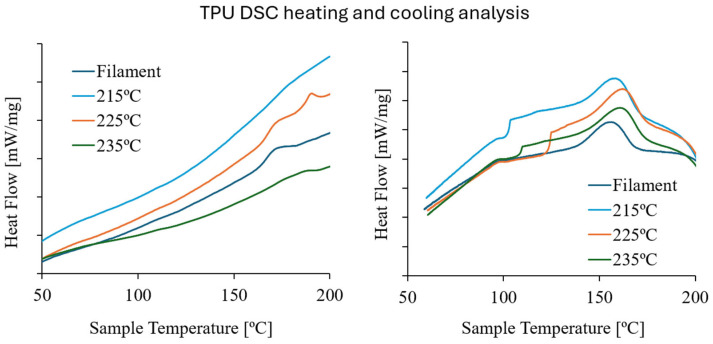
DSC curve of TPU filament and TPU samples extruded at different temperatures.

Figure 4 illustrates the thermal heating followed by cooling cycle of all TPU samples. From Figure 4, a data analysis of all relevant thermal data is presented in Table 4.

DSC results revealed that the melting temperatures (*T_m_*) and crystallization temperatures (*T_c_*) were successfully identified for all tested samples, revealing significant differences among them. *T_m_* had a variation of approximately 20 °C from filament to printed, while the crystallization temperature increased 6 °C, to 225 °C, from filament to FFF.

The results presented in Table 4 show that the *T_m_* and *T_c_* of the FFF samples are different from the TPU95A filament. The *T_m_* decrease with the samples’ extrusion temperature may result from different cooling speeds, being the lowest for the samples extruded at 215 °C, which increases the crystallinity degree of the polymer and consequently, its melting temperature. The FFF samples extruded at 235 °C present the lowest *T_m_*, probably due to their lower crystallinity degree, which contributes to a decrease in the polymer performance that can be correlated with the mechanical testing results.

#### 3.1.2. Composite Laminates Differential Thermal Analysis (DTA)/Thermogravimetric Analysis (TG)

**Figure 5:**
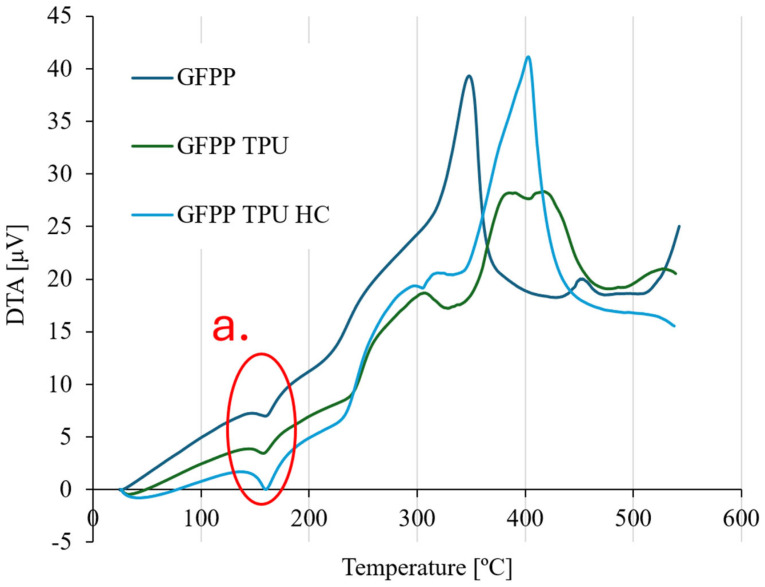
DTA curve of three types of composite laminates produced.

Figure 5 shows the DTA analysis results for three different samples of the manufactured composites, GFPP, GFPP-TPU, and GFPP-TPU-HC.

The first peaks across Figure 5 (identified by a.) correspond to the melting of the PP matrix present in all three composite samples (melting temperature at this range). The peaks at around 350 °C suggest an exothermic event. The sharp peak could be related to a decay of the PP.

The later curve peaks at around 400 °C can be attributed to the bonding between different materials (glass fibre, PP, and TPU). It is also important to highlight the differences in the degradation temperatures of PP and TPU. These variations correspond to the sharper, upward-facing peaks, which indicate exothermic reactions occurring during the degradation of the polymers. The degradation temperature of PP is lower (350 °C), while TPU degrades at a higher temperature (400 °C), which explains the distinct thermal behaviour and the shape of the curves after the peak.

TG analysis allowed for an experimental determination of the percentage (in weight) of polymers (PP only and PP+TPU, depending on the sample type) and glass fibres present in the three different composite laminate types. Table 5 presents the results of fibre and polymer mass and volume content (neglecting the existence of voids).

The results presented in Table 5 show, as expected, that the glass fibre mass fraction is higher for the GFPP sample than for the TPU-reinforced samples, and of these two samples, the TPU with a honeycomb cellular structure presents a lower polymer content than the sample produced with solid TPU.

### 3.2. Surface Morphology

**Figure 6:**
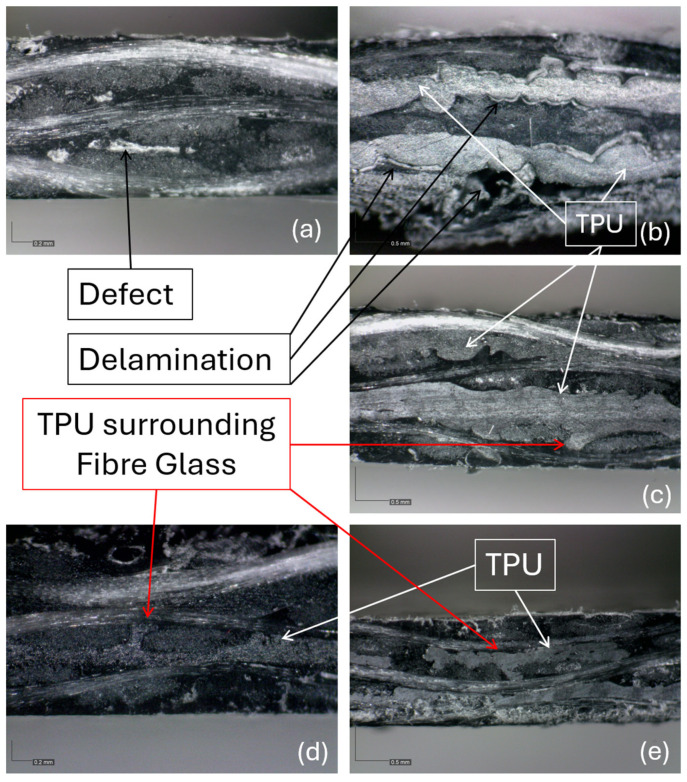
Microscopic thickness surface analysis: (**a**) GFPP laminate showing defects; (**b**) GFPP-TPU laminate with poor TPU–PP adhesion leading to delamination; (**c**) GFPP-TPU with concentrated TPU layer; (**d**) GFPP-TPU-HC with good TPU integration into the glass fibre structure; and (**e**) GFPP-TPU-HC showing uniform TPU dispersion and strong interfacial adhesion.

Before and after three-point bending characterization, all laminate specimens were examined using optical microscopy. Representative cross-sectional microscopic images are presented in Figure 6, highlighting key observed features. All microscope images were captured with an OPTIKA microscope equipped with a Dino-Eye Edge digital camera.

In some GFPP laminates without TPU reinforcement (Figure 6a), defects such as voids between layers were observed. In contrast, TPU-reinforced laminates (GFPP-TPU and GFPP-TPU-HC) did not exhibit these voids. However, some TPU-reinforced specimens showed regions with TPU accumulation (Figure 6c) or poor adhesion between the TPU and the PP matrix. In such cases, delamination occurred at the TPU–PP interface during three-point bending tests, as shown in Figure 6b.

Specimens with good interface adhesion between TPU and PP exhibited no delamination. Microscopic analysis revealed that in these cases TPU effectively surrounded and even partially embedded into the glass fibre bundles (Figure 6c–e), indicating a strong bonding. Notably, the honeycomb structure appeared to enhance the uniformity and distribution of the TPU within the laminate, promoting better integration with the PP matrix. These observations are consistent with the bending test results, which showed no visible delamination in any of the GFPP-TPU-HC specimens.

### 3.3. Mechanical Testing Results

#### 3.3.1. Tensile Testing

**Figure 7:**
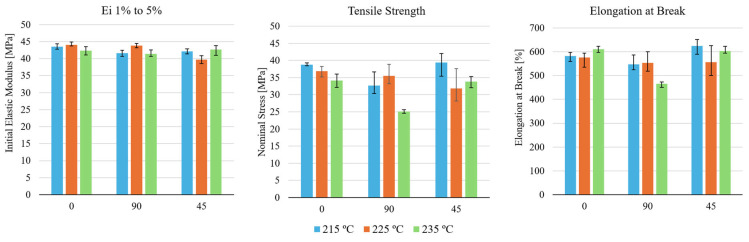
TPU bulk tensile test results for initial elastic modulus (Ei 1% to 5%), tensile strength, and elongation at break.

As previously described in Section 2.2, both bulk and cellular structure TPU specimens were submitted to tensile testing. The results of the main mechanical properties for bulk specimens are presented and compared in Figure 7.

The results reveal the expected anisotropic behaviour inherent in the FFF manufacturing process. The most pronounced differences appear in parameters related to plastic deformation and failure—tensile strength and elongation at break. In contrast, the initial elastic modulus shows minimal variation, being in general higher at an extrusion temperature of 225 °C. These findings indicate that filament and layer fusion did not introduce significant anisotropy in the elastic region, regardless of build orientation or extrusion temperature. However, anisotropy becomes much more evident in the plastic and fracture regimes.

Transverse specimens (90°) exhibited the lowest tensile strength and elongation at break, clearly demonstrating this effect. In 90° build orientation, filament and fusion regions experience an iso-stress condition where both are subjected to the same stress. These weld regions often contain defects, voids, and altered polymer crystallinity, all of which contribute to premature failure and reduced mechanical performance. An equivalent conclusion was taken by Sardinha [22] where voids in FFF TPU are aligned with the infill filament deposition direction. This translated to transverse TPU specimens presenting lower tensile strength.

Following this concept, the extrusion temperature proved a notable influence on the mechanical performance of the 90° and 45° specimens, particularly in terms of tensile strength and elongation at break. The most significant change was observed in 90° specimens, where tensile strength decreased by 29.4% when the extrusion temperature increased from 225 °C to 235 °C. Such might indicate that higher temperatures have poorer quality filament welded regions or lead to a polymer with a lower crystallinity degree as a result of its higher cooling speed during the extrusion due to the higher temperature gradient between the extruded polymer and the heating bed (at 60°). Several studies have shown that increasing extrusion temperature generally reduces void size and frequency, likely due to decreased melt viscosity and improved interlayer and filament fusion [22,31]. It is therefore expected that higher temperatures would result in improved mechanical properties as perceived by the referenced studies. The unforeseen result could therefore be associated with polymeric temperature gradients that result in a lower crystallinity degree as proven by the DTA results.

When comparing the results with the manufacturer mechanical properties data shown in Table 1, there is generally a lower value. The lowest to highest differences in each mechanical property are as follows: 5.6% to 29.7% in elongation at break; 10.9% to 43.2% in tensile strength; and 9% to 17.9% for the initial elastic modulus. Again, the plastic deformation parameters present the biggest variation and difference.

**Figure 8:**
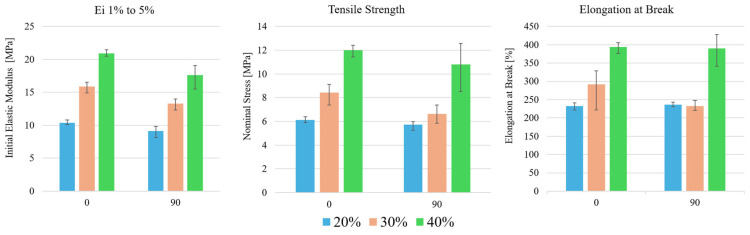
TPU honeycomb tensile test results for initial elastic modulus (Ei 1% to 5%); tensile strength; and elongation at break.

The tensile testing results of the honeycomb geometry TPU specimens are presented and compared in Figure 8.

As expected, all three computed parameters increase in value with the increase in relative density. A general decrease in mechanical properties is also visible between 0° and 90°. Additionally, a generally higher variability in results was obtained with 90° aligned structures. Like bulk specimens, the initial elastic modulus had the lowest variability between the three computed parameters.

**Figure 9:**
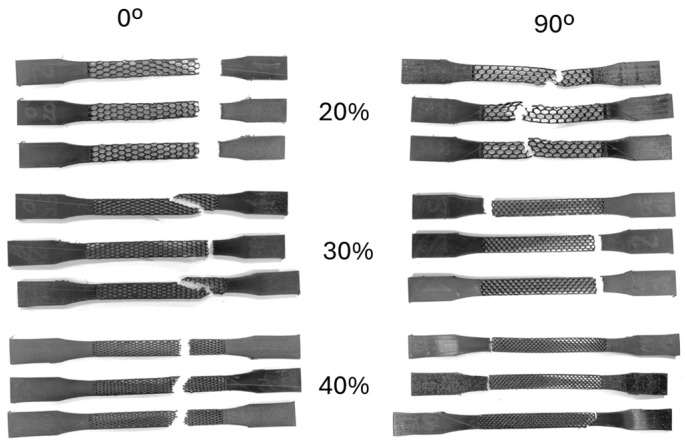
Tensile fracture of honeycomb TPU specimens.

The specimens’ fractured region was between the honeycomb and bulk section and in the honeycomb itself. A significant difference between the 0° and 90° fracture location was visible. While 0° specimens transitioned from the connection region towards the cellular structure as the relative density increased, the 90° specimens presented the opposite behaviour. Figure 9 shows fractured specimens. The relative density with both types of fracture results in a higher variability in the plastic deformation mechanical properties, having a possible reduction of when it transitions from cellular to the cellular-bulk region. This is visible when comparing 90° cellular structure between 20% and 30%, where there is even a reduction on elongation at break.

**Figure 10:**
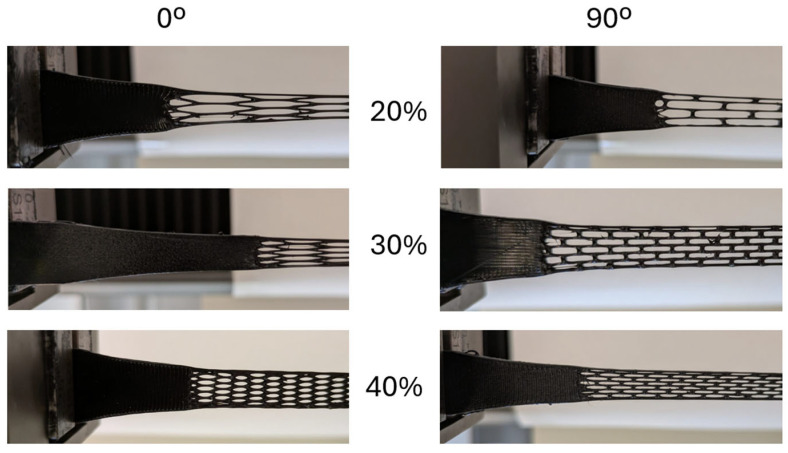
Cellular-bulk region for all types of honeycomb TPU specimens at a high deformation instant.

The failure region change appears to be associated with combining the factors of the structure relative density, the cell deformed shape at high deformation, and how the cells are connected to the bulk. It is possible to perceive how the load transmission in the cellular-bulk may affect the change in fracture location when observing the cellular-bulk region at high deformation, as shown in Figure 10.

In 0° specimens with 20% relative density, a pronounced “C-shape” is visible, which indicates asymmetric deformation where the outermost cells carry most of the tensile load. As the cell infill increases, the curvature diminishes, suggesting a more uniform load distribution which coincides with all fractures shifting to the honeycomb rather than the interface. In contrast, 90° specimens do not display the C-shaped distortion. The honeycomb cells align with the tensile axis at high deformation, forming straighter load paths and promoting more balanced load distribution from bulk to cellular. This likely explains why 90° specimens at 20% showed greater elongation at break than their 0° counterparts.

Even with the high deformation alignment, the 90° honeycomb fracture shifted to the cellular-bulk regions at higher relative densities. This shift may be due to weaker bonding quality due to the printing sequence, or stress concentration when the cell ends at specific connection regions. In the 0° and 20% specimens, fracture consistently initiated on the same interface where the contact area between honeycomb and bulk was visibly reduced. In the remaining specimens, the connection was made approximately equal, resulting in alternate fractures between cellular-bulk sides.

Future FFF cellular tensile specimen designs must be studied to reduce fracture in the bulk to cellular region, allowing for more cohesive plastic deformation and fracture characterization.

**Figure 11:**
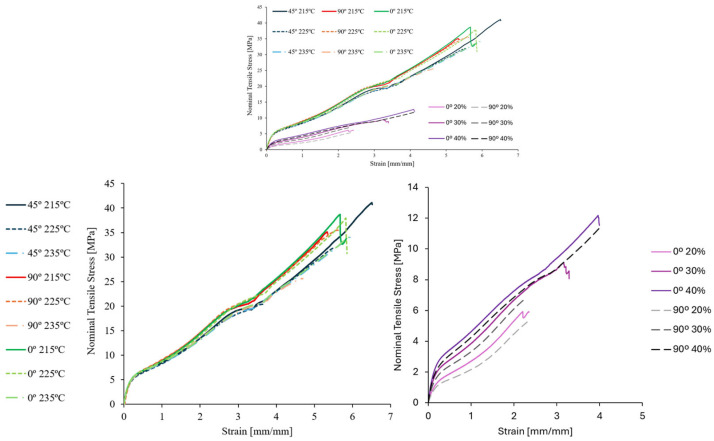
Nominal stress–strain diagram for bulk (**left**) and honeycomb (**right**) TPU tensile test results.

**Figure 12:**
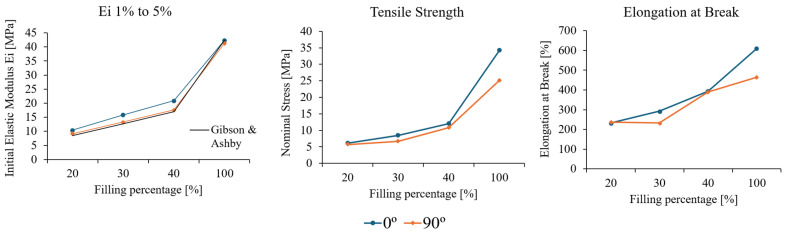
Initial elastic modulus (Ei 1% to 5%), tensile strength, and elongation at break in relation to the relative density percentage.

The cellular specimens’ results were then compared with the bulk specimens. Representative stress–strain results between all TPU tensile-tested specimens, bulk and honeycomb, are presented in Figure 11. Figure 12 correlates the percentage of relative density with the three main parameters under discussion.

Figure 12 shows a clear and predictable increasing trend across the three properties. The results demonstrate good agreement with the analytical solution for the effective moduli determined by Ashby and Gibson [34] considering the coefficients *C_1_* and *n* equal to 1. The 0° aligned cellular structure exhibits higher moduli than predicted, highlighting the orthotropic behaviour of the honeycomb arrangement.

#### 3.3.2. Three-Point Bending Test

**Figure 13:**
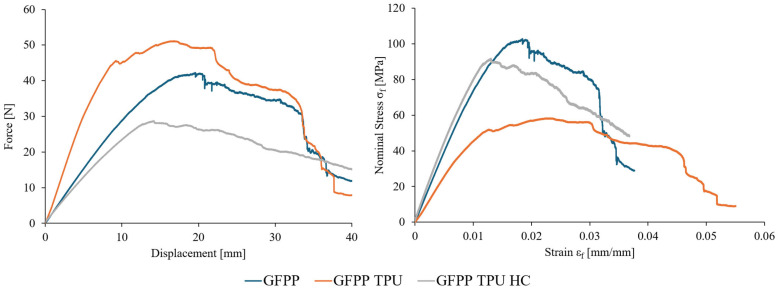
Representative three-point bending nominal stress–strain and force–displacement diagrams for the three tested laminated composites.

Following the TPU tensile characterization, all three composite laminates were subjected to three-point bending tests. Representative stress–strain and force–displacement curves for each laminate are presented in Figure 13.

A direct analysis of the Figure 13 curves reveals clear distinctions between the three laminates. Baseline GFPP has the highest bending strength; however, once plastic deformation is reached it exhibits an uneven loss of strength with sudden force drops, attributed to fibre and inter-ply failure. The bulk TPU-reinforced laminates (GFPP-TPU) sustain the highest deformation prior to strength loss. This is attributed to the shear deformation ability of the inter ply TPU layers. Conversely, GFPP-TPU displayed the lowest flexural modulus and bending strength, and several specimens experienced sudden strength drops (similar but not as distinctive as GFPP) predominantly associated with inter-ply failure (confirmed by microscopic analysis, Figure 6d).

GFPP-TPU-HC laminates exhibited the most interesting results. Their plastic deformation was uniform, with a gradual and progressive loss of strength. Apart from one exception, no delamination or fibre breakage was observed under post-test microscopy. Moreover, specimens presented high recoverability, having almost returned to their original shape after testing.

**Figure 14:**
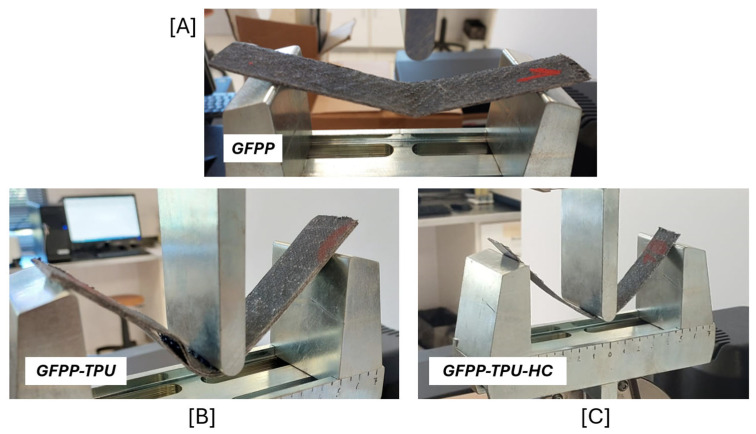
Three-point bending tests: (**A**) GFPP; (**B**) GFPP-TPU; and (**C**) GFPP-TPU-HC.

Figure 14 illustrates failure examples of all three laminates under the three-point bending test: fibre and layer breakage in GFPP (Figure 14A), pronounced delamination in GFPP-TPU (Figure 14B), and no visible damage in GFPP-TPU-HC (Figure 14C). These visual results align closely with the mechanical patterns concluded from Figure 13.

**Figure 15:**
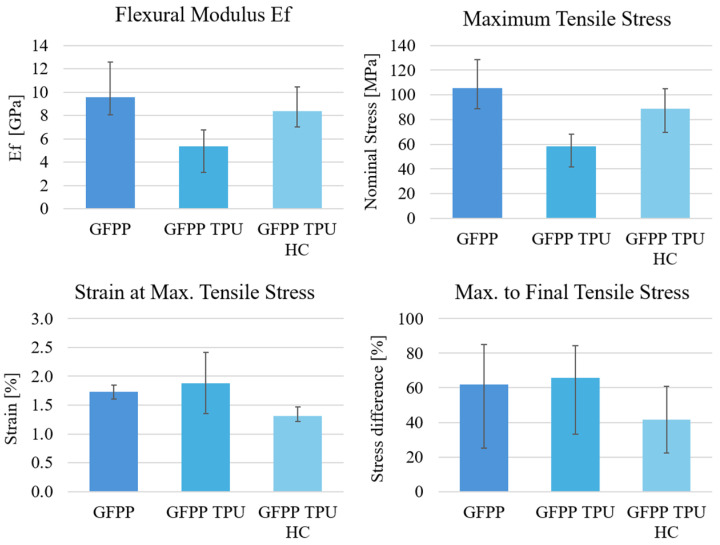
Flexural modulus (E_f_); maximum tensile strength; strain at maximum tensile stress; and the ratio between the maximum and the final tensile stress for the laminated composites produced.

From strain–stress diagrams, four mechanical parameters were computed and compared across the three laminates: flexural modulus (E_f_), maximum tensile stress, strain at maximum tensile stress, and the ratio between the maximum-to-final tensile stress. All computed results are summarized in Figure 15.

Previous studies on the three-point bending of GFPP laminates without interlayer reinforcement report flexural moduli and strength in agreement with the values presented in Figure 15 [35,36,37]. Balaji Ragupathi and Frank Balle [35] observed interlaminar failure in GFPP laminates even after ultrasonic reconsolidation highlighting the inherent weakness of the interlaminar region. Furthermore, both Ragupathi [35] and Michael I. Okereke [37] reported drops in flexural strength after peak load in 0/90 laminates, a behaviour also observed in the present study.

The results shown in Figure 15 reinforce the conclusion drawn from the Figure 13 diagrams, highlighting the benefits of applying honeycomb TPU interlayer reinforcements. A clear trend is observed towards a lower maximum-to-final tensile stress ratio without a significant loss of flexural modulus. This indicates an improved ability of the laminate to sustain high deformation without delamination and fibre breakage.

## 4. Conclusions

This study explored the use of additively manufactured thermoplastic polyurethane interlayers, manufactured by FFF in bulk and in honeycomb cellular structures, to enhance the mechanical performance of glass fibre-reinforced polypropylene laminates. Mechanical, thermal, and morphological analyses demonstrated the potential of TPU as an effective interlayer toughening material when integrated through vacuum bag oven curing.

The tensile testing of printed TPU confirmed its elastomeric characteristic, with high deformability and recoverability, supporting its role as a shear-compliant interlayer. Printing parameters affected mostly plastic and fracture characteristics.

All conducted measurements and experiences highlighted significant differences between the three laminated composite configurations. From the performed analysis, the study concluded the following:

- Strong adhesion between GFPP and TPU layers was achieved without voids.
- Honeycomb layers allowed for more cohesive laminate manufacture.
- Bulk TPU layers were prone to delamination and significantly reduced bending stiffness. In some specimens, good adhesion was achieved, meaning the manufacturing process can be optimized.
- Laminates with a honeycomb TPU interlayer exhibited the highest resistance to delamination and fibre breakage, attributed to improved adhesion between the TPU and the polypropylene matrix.
- Honeycomb reinforcement also enhanced bending deformation capacity and overall ductility, displayed by the high elastic recovery and the lower maximum-to-final nominal stress ratio.

These findings confirm TPU as a viable interlayer toughening strategy for thermoplastic laminates. Future work should continue to explore and optimize the integration of elastomers for multifunctional applications, leveraging their inherent properties such as vibration damping, impact resistance, and high elastic recovery. This study also demonstrates that FFF can serve as more than a prototyping tool, providing a powerful means of tailoring interlayer architectures and enhancing the multifunctional performance of structural composites. By exploiting the design freedom of additive manufacturing, future research can extend interlayer engineering toward adaptive, energy-absorbing, and smart material systems.

## Figures and Tables

**Table 1:** Mechanical and thermal properties of TPU95A Ultrafuse filament according to BASF manufacturer [26].

Shore Hardness	Density [kg/m^3^]	Tensile Strength [MPa]	Elongation at Break [%]	Elastic Modulus [MPa]
92A	1149	44.2	661	48.4
**Glass Transition** **Temperature [°C]**		**Melting Temperature [** **°** **C]**	**Recommended Nozzle** **Temperature [°C]**	
−25		144	210–230	

**Table 2:** COMFIL 30028-17 WG1-PP-700 black weave twill 2/2 thermoplastic prepreg properties according to the manufacturer [32].

Consolidation Temperature [°C]	Glass Fibre Content by Volume [%]	Nominal Weight [g/m^2^]	Density [kg/m^3^]	Thickness of Fully Consolidated Ply [mm]
190–230	35%	700	1560	0.47

**Table 3:** Manufactured laminates, abbreviations, and layer sequence.

Composite Laminate	Abbreviation	Layer Sequence
Baseline glass fibre prepreg with polypropylene	GFPP	[(0/90)_3_]
GFPP with solid TPU layers	GFPP-TPU	[0/90/TPU/0/90/TPU/0/90]
GFPP with honeycomb-structured TPU layers	GFPP-TPU-HC	[0/90/TPU-HC/0/90/TPU-HC/0/90]

**Table 4:** DSC melting temperature of TPU filament and samples produced at different temperatures.

Tested Material	Melting Temperature *T_m_*	Crystallization Temperature *T_c_*
Filament TPU95A Ultrafuse	172.1 °C	155.1 °C
FFF extruded at 215 °C	191.0 °C	157.1 °C
FFF extruded at 225 °C	190.1 °C	161.6 °C
FFF extruded at 235 °C	185.1 °C	159.5 °C

**Table 5:** Fibre glass and polymer mass and volume fractions in composite laminate specimens.

	Mass Fraction [%]		Volume Fraction [%]	
Samples	Fibre Glass	Polymer	Fibre Glass	Polymer
GFPP	62.5	37.5	37.7	62.3
GFPP-TPU	51.4	48.6	25.9	74.1
GFPP-TPU-HC	56.9	43.1	35.4	64.6

## Data Availability

The original contributions presented in this study are included in the article. Further inquiries can be directed to the corresponding author.
